# *Ciranda*, a circle of encounter: Reflections on a decolonial pedagogical activity on human rights discourses with Latinx students

**DOI:** 10.1057/s41276-023-00417-3

**Published:** 2023-04-19

**Authors:** Jesica Siham Fernández, James Moura Ferreira

**Affiliations:** 1 Santa Clara University, Santa Clara, USA; 2 University for International Integration of the Afro-Brazilian Lusophony/Federal University of Ceará, Redencao, Brazil

In the United States, approximately one in five college students are of Latinx^1^ descent or identify as Latinx. The representation of Latinx in higher education has been steadily increasing in recent years. However, the trend slightly decreased during the 2021 academic year because of the COVID-19 pandemic. Recent reports indicate that some Latinx students are postponing higher education to support and care for their families (Marshall et al. 2021). In the context of providing Latinx students with meaningful learning opportunities that foster a sense of community, alongside a critical consciousness, we facilitated a decolonial pedagogical activity to help members of the Latinx Student Union (LSU) reflect on human rights discourses in the United States. Higher education institutions, while purporting the development of citizen leaders of conscience and democratic engagement, are often experienced by Latinx as oppressive disenfranchising spaces because such settings do not actually enact equity, diversity, and inclusion (Bell 2018).

As a response to these conditions, we implemented an intervention, the Circle of Encounter decolonial pedagogical activity, called *ciranda*, with the Latinx Student Union (LSU) at a private Jesuit institution in the Silicon Valley (California, United States). The activity sought to cultivate a critical consciousness about the implications of racist nativism, structural violence, whiteness/white supremacy, and human rights as these manifested in their lives, families, and communities. In this way, our goal was to facilitate a space for Latinx students to engage with these topics, and to reflect on the significance or meanings of human rights.

In this *reflexión pedagógica*, we offer a brief description of the *ciranda*, or Circle of Encounter activity. We reflect on how we facilitated the *ciranda,* which has roots in Latin American popular education, decolonial pedagogy, embodied experiential learning, and social movements. Additionally, we reflect on our process of leading the activity and learning from Latinx students who challenged sociocultural political discourses of human rights. We present a decolonial pedagogical activity, the *ciranda*, that contrasts the banking model of education, which is a form of disembodied learning (Watkins et al. 2018). We demonstrate how Latinx students conceptualized notions of human rights in relation to the experiences of Latinx in the United States, specifically immigrant and undocumented families. Our *reflexión pedagógica* illustrates our practice and commitment to cultivating Latinx students’ critical consciousness through reflexive dialogues grounded in a decolonial pedagogy aligned with Latinx studies. Our process of cultivating critical consciousness through reflexive dialogues and embodied learning via dance is characterized by the *ciranda*. We offer this activity as a strategy for educators desiring to facilitate learning opportunities for students that are grounded in a Latin American decolonial pedagogy.

## Conceptualizing human rights

Institutionalized, cultural, and social practices within the United States operate and are structured to violate the human rights of Latinx immigrants, especially undocumented and/or mixed-status immigrants (Langhout and Vaccarino‐Ruiz 2021). Structural and symbolic violence have a colonial basis for these violations. Structural violence, while informed by symbolic violence, is defined as the social and cultural systems, along with the institutions, practices, and relationships by which marginalization, oppression, and dehumanization is reproduced and sustained against people of color since the colonial period (Dutta et al. 2016). United States immigration policy reifies practices of direct violence against Latinx immigrants, in what Sampaio (2015) describes as the terrorizing of Latinx families, followed by the criminalization of immigrants (Menjívar 2013), who are problematically framed as likely to commit more crimes than United States citizens (Provine and Doty. 2011). The dominant ideology stigmatizes marginalized groups at the same time that it legitimizes coloniality through acts of violence that are dehumanizing (Martín-Baró 1994).

Reparation strategies that resist such violations and violence against Latin American populations are necessary, especially in neoliberal contexts such as that of the United States, as well as within institutions that are accountable for developing concrete reparations and humanizing policies yet are reluctant to do so (Crosby and Lykes 2019). Conceptions of human rights must be contextualized, embodied, enacted and applied to attend to the unique circumstances of groups affected by these systems (Menjívar 2013). Reparation strategies must meet the needs and concerns of such communities (Lykes and van der Merwe 2019). To carry out the process of reparations for historic and contemporary acts of violence, it is necessary to understand reality critically, and to act with a focus on changing oppressive structures and systems. The starting point of this process is often challenging, interrogating and deconstructing what is known through a decolonial pedagogy that involves unlearning and relearning (Freire 1974). Toward that end, we draw on our experiences in leading a Circle of Encounter activity with Latinx students, and we offer our reflections on students’ perspectives on human rights as they engaged in the *ciranda*.

## A brief note on our positionalities

As colleagues and collaborators on various projects, as well as teacher-scholars-activists, we see our teaching and research as integrated with our pedagogy and activism. We engage in liberation and critical psychologies from the majority world that build on the work of Martin-Baró and Freire, and are also grounded in the decolonial feminist writings of María Lugones and Gloria Anzaldúa. As equally contributing authors, we recognize that, while our values and ethics as scholars are in resonance, we are differentially positioned along dimensions of race/ethnicity, gender, sexuality, and lived experience; thus we offer a brief note on our positionalities.

Jesica is a cisgender Chicana, born in México and raised and schooled in the United States, who embodies the common expression of being “neither here, nor there”; too Mexican for the “Americans,” too Americanized for the Mexicanos. As such, Jesica lives the nuances, complexities, and contradictions of being Latinx in an ever-changing United States sociopolitical climate. James identifies as a South American white male from a working-class family. As a faculty member at a public Brazilian university, he visited the United States as Fulbright Scholar in the fall of 2021 to continue his teaching and pursue comparative research on college students’ perspectives on human rights and violence in United States universities. Our shared commitment to decolonize the university brought us together to develop and facilitate the *ciranda*, or Circle of Encounter.

## *Ciranda* activity, a circle of encounter with Latinx students

### Ciranda’s purpose, elements, and structure

The Circle of Encounter is a *ciranda,* a decolonial pedagogical activity that was first developed in the context of Latin American popular education. In addition to being a pedagogical approach, it is also a community-mobilizing or movement-building strategy (Góis 2005). As a decolonial pedagogical activity developed by Brazilian community psychologist Cezar Wagner, the Circle of Encounter (Moura et al. 2016) shares some similarities with Freire’s technique of Culture Circle (Freirw 1979), and Rolando Toro’s Biodanza exercises (2005). The *ciranda* fosters a relationally embodied space with the potential to strengthen relationships among its participants. Creating an atmosphere of respect, affection, and proximity to others is essential for developing an embodied experiential learning experience. Because the *ciranda* involves sharing personal and collective experiences it also has the potential to surface emotions, some of which may be challenging to articulate, such as those described by Latinx students of immigrant and undocumented families in the United States (Moura et al. 2016).

We began the activity by establishing an atmosphere of care and respect for one another, especially among students. We pursued this through play, humility, and curiosity in the form of an ice-breaker or community-building game that involved Latinx students introducing themselves in a creative way. We asked Latinx students to state their names and perform or act out a movement that could produce a visual to be associated with their name. For example, a student shared: *My name is Ana, and I like to dance* (making a dance movement). Each student stated their name, and an activity they enjoyed, which they demonstrated with a movement. This was our initial step toward de-mechanizing our bodies via movements or a playful exercise that was purposeful about creating community (Góis 2005). Fostering Latinx students’ sense of play through humility and curiosity was an essential component in our process for facilitating the *ciranda* as an activity toward promoting critical consciousness (Friere 1984). The ice-breaker we led aligned with Maria Lugones’s notion of loving playfulness because it involved movement, embodiment, and experience. It also aligned with Gloria Anzaldúa’s notion of “making faces,” which is recognizing our individual and collective expressions, emotions, and vulnerabilities alongside the joy (Lugones 1987). The embodied affectivities of the ice-breaker subsequently allowed us to transition onto the *ciranda* activity (see Image 1).

**Figure:**
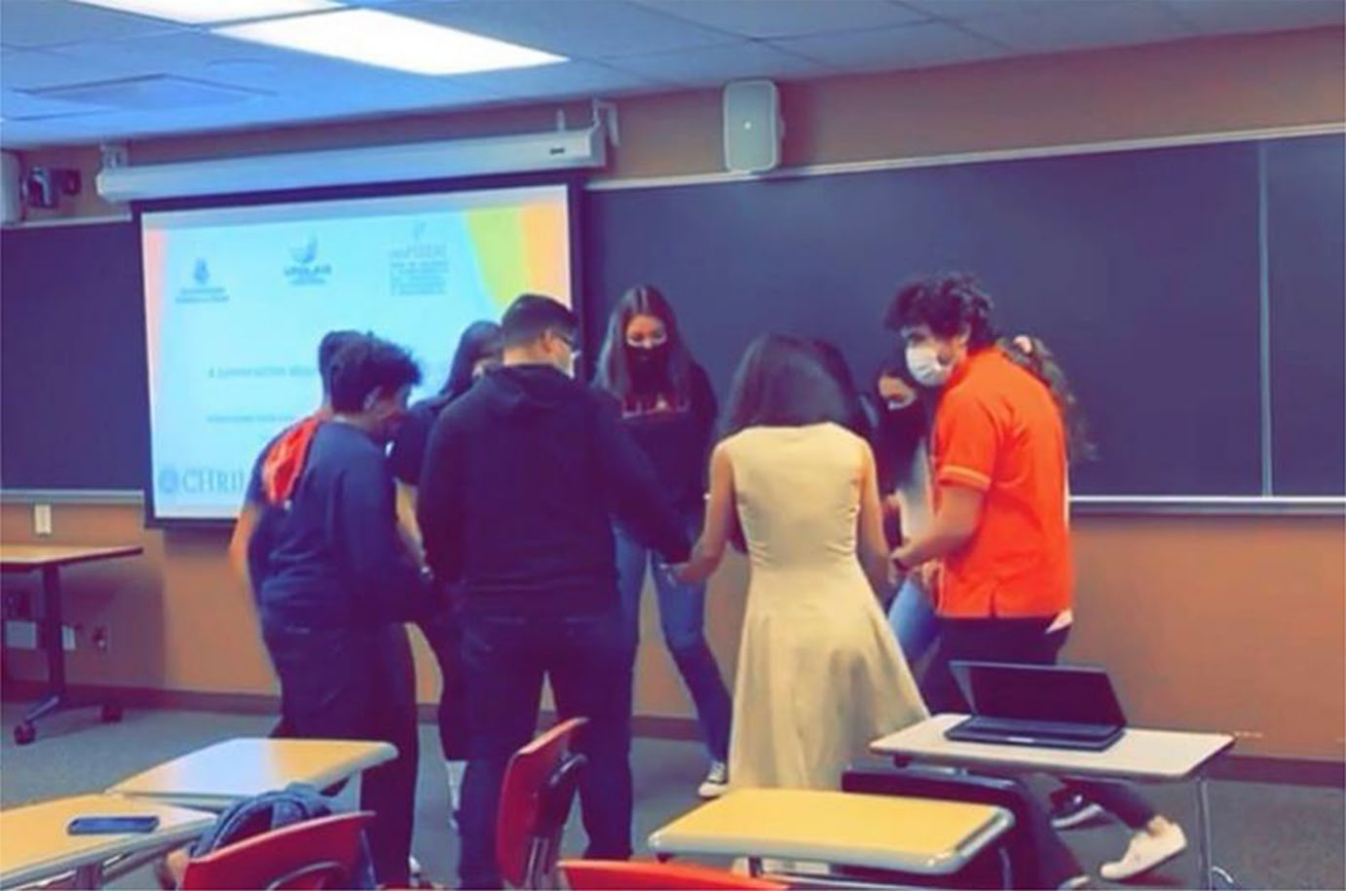
A photograph of the *ciranda* activity in practice

The *ciranda* activity began with us forming a circle in the center of the classroom. We were intentional in striving to create a space of agency, connectivity, and comfort, as well as safety. We facilitated the *ciranda* in an indoor classroom space where we abided by COVID-19 protocols of wearing face masks. Students were encouraged to consider what was within their comfort and capacities, and to join or stop their participation at any moment. While we were hesitant about holding hands, because of social distancing protocols, we invited students to hold hands with each other nonetheless. One of the facilitators asked the students to start a joint circular movement, making a *ciranda* to the song “Eu sou Lia minha ciranda preta” by the Brazilian Black singer Lia de Itamaraca. In *ciranda*, rhythmic music is used to perform circular movements with participants; each person steps and swings their arms synchronously to the beat and flow of the music (Lugones 1987). The students began to dance the *ciranda* collectively in a circular form while holding hands. Latinx students’ movements were initially loose and without rhythm; however, as we were joined by our hands, we began to eventually flow and move as one.

Via synchronized relational movements, *ciranda* participants began to identify themselves as a part of a collective, or in relation with others (Moura et al. 2016). Each participant in the *ciranda* had their way of moving. There were no judgments about the forms of expression (Toro 2005). In this way, we created an atmosphere of respect, horizontality, and recognition of the ontological vocation of being human—that is, of being more than what society has prescribed some people to be: workers, students, consumers, subhumans (Freire 1984). The connection via movement served as the foundation for the reflexive dialogues on difficult topics that would involve deconstructing violence. Because the body concretely felt this connection to others in the circle, we began to create an integrative experience that provided an embodied learning process as a community (Ciofalo 2021).

As we continued to move, some Latinx students took the initiative to shift, alternate, or introduce new movements, while others made the rhythmic movements. One of the facilitators placed a paper with the words “Human Rights” in the center of the *ciranda*. The word, and several others generated thereafter, such as “Violence” and “Reparations,” served to deepen collective reflexivity via movement. While no conversations or dialogues took place at this moment, the presence of these words at the center of the circle, the music, and our collective movement compelled us to ground ourselves. Although some words appeared abstract, the words “human” and “rights” were felt in the body in ways that illustrated Cherrie Moraga’s notion of a “theory in the flesh” (Anzaldúa and Moraga 1981). In this way, *ciranda* builds on the relationship of movement, body, and emotion with a specific theme, and leads to a process of embodied reflexivity. Thus, as we continued to move, one of the facilitators asked the Latinx students to keep dancing in a circular manner while we added more words at the center of the *ciranda*. When the music ended, the Latinx students expressed feeling more integrated and energized. As a decolonial pedagogical activity, the *ciranda* is a corporally integrated relational learning process that helped deepen Latinx students’ critical consciousness about social realities, inequities, and injustices (Escobar 2016; Góis 2012).

After the *ciranda*, we invited Latinx students to gather around and form a circle in the rearranged classroom desks. The feeling of connectivity facilitated by the *ciranda* helped us begin to reflect and engage in dialogues that led us to bear witness to our shared struggles. This subsequently led us to unearth our individual and collective pain or anger at the injustices and dehumanization we experience; the words shared at the center of the *ciranda* evoked such reflections and the meaning or absence of human rights in lives. As facilitators, we asked Latinx students to share their reflections and experiences related to human rights. To deepen their reflexive dialogues, we encouraged Latinx students to share their feelings and supported them in opening up to share their reflection on human rights (Freire 1979).

The purpose of *ciranda* is to create a space to exchange knowledge and movements, thereby facilitating processes of embodied consciousness and critical reflexivity (Góis 2005). We have offered a description of our implementation approach, which we share as an invitation for educators to consider adapting with students or youth within their unique contexts. Educators should approach each student as capable of facilitating and co-leading the experience relationally and working collaboratively in mutual accompaniment. In the discussion that follows, we reflect on the dialogues and experiences shared by Latinx students who participated in the *ciranda*.

## Latinx students’ reflections on human rights after the *ciranda*

In the *ciranda* we facilitated with Latinx students, human rights violations were discussed among them as they saw these manifest in their families and communities, as well as in their lives. Building an atmosphere of community, belonging, and horizontality through *ciranda* allowed students to share their life experiences, as well as critical, collective, and embodied learning (Moura et al. 2016). Among the first remarks offered by Latinx students was the naming of how, in the United States. some Latinx have traumatic experiences with violence and discrimination. Indeed, students were quick to name intersectional experiences of oppression, institutional racism, discrimination, criminalization, and dehumanization rooted in white supremacy and coloniality. Because of the *ciranda*, Latinx students were more likely to feel connected to each other—a connection that was cultivated through movement or dance. Students were more keen to express a sense of vulnerability and transparency in voicing their stories because the *ciranda* encouraged reciprocity, collaboration, and intimacy through movement and touch (James and Lorenz 2021).

The intimate safe space of the *ciranda* helped one student share their fears and anxieties with seeking out health-care services and resources in the context of COVID-19 because of their undocumented status. Similarly, other Latinx students shared that, because of the inadequate support their undocumented families receive, concepts of human rights related to democracy, societal well-being, and respect for freedom and dignity must be questioned. Latinx students noted that because they and their families experienced a reality permeated by rights violations, dehumanization, and systemic violence, human rights were aspirational and inadequately put into practice. Relatedly, Latinx students understood that institutions can reproduce systems of power even within spaces that are purported to be inclusive, such as the university. The movement Latinx students engaged in prior to opening up about their experiences prepared them to see themselves as beings with bodies in relation to each other, and as part of a whole (Escobar 2016). Seeing themselves as belonging to a community rather than as individuals with burdens and struggles to carry alone, Latinx students were more likely to question the realities of their lives (Watkins et al. 2018).

Further, Latinx students described examples of human rights violations that led them to be critical of human rights discourses. Some students stated that human rights discourses are rooted in Eurocentric perspectives that overlook the histories of coloniality and colonialism, and their contemporary manifestations in the lives of their families in the United States. Reflecting on the *ciranda*, they remarked that there are few opportunities for them to learn about their community histories and struggles. Similarly, they expressed that there is a need to engage in practices, like dance and artistic expression, that are rooted in community cultural ways of being. One student, for instance, stated, “Why do we need human rights to tell other people that they should treat other humans right, with dignity and respect? If we treated each other with dignity—as human beings—we wouldn’t need human rights.” Generally, human rights were viewed as too individualistic, which was another critique students offered (Ignatieff 2003). In other words, individual rights and freedoms, rather than collective human rights, were seen as most valued or important. One Latinx student in particular described this as dancing alone versus dancing as part of a collective.

Reflecting on their collective experiences, some Latinx students shared why their parents emigrated to the United States. One student disclosed that their family experienced extreme violence and poverty in their home country, and sought to pursue a more dignified life by emigrating. However, that pursuit was more challenging than imagined because of anti-immigrant discourses and racism. Similarly, other Latinx students described the reality of poverty and violence in their home countries to the geopolitical actions or interventions by the United States in foreign affairs—both in historic and contemporary contexts. Such reflections resonate with Martin-Baró’s writings that purport that Latin America’s problems are maintained by global geopolitics, in which the United States has had significant influence (Martín-Baró 1985). In moving toward dialogues of radical hope and action, some students pointed out that reparations are necessary, and these must have concrete actions as outcomes. Through their reflexive dialogues, Latinx students expressed their critiques of human rights alongside the varied forms of violence they witness and experience. Latinx students, moreover, pointed to different strategies for repairing historic inequities, injustices, and structural violence. The embodied experiential learning that was facilitated through the *ciranda* was considered by some Latinx students as practical action or strategy to begin to question knowledge, power, and systems of oppression that divide and fragment (Moura et al. 2016).

Latinx students’ experiences in *ciranda*, together with their perceptions and feelings during and after the activity, culminated in their shared expressions of gratitude for the opportunity to participate in the Circle of Encounter*—*a learning space that helped deepen their understanding of human rights violations and reparations for communities. Latinx students named the importance of fostering spaces of reflection where conversations on human rights could take place. Such a dialogue was possible because the *ciranda* activity we facilitated helped us and Latinx students engage in constructing alternative, collective realities through movement, embodiment, and reflexivity.

## Ciranda as a decolonial pedagogical activity

As a decolonial pedagogical activity, the *ciranda* facilitates critically reflexive dialogues on social problems by bringing people together through feeling and movement in community. When people are called to connect their lived experiences with their feelings and emotions, as well as their bodies and movement in relation with others, learning becomes an embodied experiential and relational process (Escobar 2016). As facilitators, we have summarized some Latinx students' reflections, which point to rights violations that must and can be changed through intersectional solidarities in action. We highlight that the *ciranda* is the recognition or consciousness of collective action as foundational to actualizing transformative justice in the form of reparations. In this way, we closed our reflexive dialogues with Latinx students with another *ciranda,* moving to the song “Todo Cambia” by Mercedes Sosa. The songs chosen for the *ciranda* must have integrative sounds that favor fluid movements, and their lyrics must hold messages or meaning that can be linked to meaningful topics for dialogue (Góis 2005). Mercedes Sosa’s music brings meaning to collective change, while focusing on the importance of unity. The students were moved by the synchronous movement that quickly became rhythmic with the music, and the reflections shared by the facilitators as the *ciranda* was performed.

The reflexive dialogues that we engaged in our debriefing, after the *ciranda* was performed, characterizes the decolonial pedagogy that is so central to Latinx studies, and that Latinx students are most drawn to engage in as a mode of learning that is rooted in an ethic of *comunidad* and authentic care. In the words of Parker Palmer, as cited by bell hooks in *Teaching Community: A Pedagogy of Hope*, “Here, the act of knowing is an act of love, the act of entering and embracing the reality of the other, of allowing the other to enter and embrace our own. In such knowledge, we know and are known as members of one community” (hook 2003, p. 8). This process of entering the “life of others” was the cornerstone of the writings by Anzaldúa and Moraga in their groundbreaking anthology *This Bridge Called My Back* (Anzaldúa and Moraga 1981). Relatedly, it informs the work of Lugones, who urged us to engage in what she described as playfulness or accompaniment with those in the struggle for liberation. Lugones writes that “while playful we have not abandoned ourselves, nor are we stuck in any particular ‘world.’ We are there creatively. We are not passive” (Lugones 1987, p. 17). It is with this orientation, toward fostering spaces of hope, possibility, imagination, and playfulness, that we approached the implementation of the *ciranda*.

## Closing reflections and recommendations for educators

Freire (1979) points out that it is necessary to comprehend reality in a questioning way to develop a critical consciousness. The group experience of the *ciranda* therefore served as a decolonial pedagogy for developing knowledge and understanding, at the same time as Latinx students challenged and deconstructed discourses on human rights that overlook the experiences and desires of communities affected by these systems who continued to live on the margins (Ciofalo 2021). Radical hope alongside collective action were named as strategies for imagining other possible worlds (Ginwright 2010). Such remarks reflect Freire’s view, as well as those of Anzaldúa, Moraga, and Lugones, that we must imagine a utopia to pursue a more just reality (Freire 1984). In describing the *ciranda* activity, it is our hope that educators will be encouraged to consider how they can incorporate art, music, performance, movement, and affect into their pedagogy, especially in relation to difficult topics associated with the coloniality of power. We therefore have offered this *reflexión pedagógica* as an invitation to encourage or inspire educators to reflect on the role of the body, affect, emotions, or embodied subjectivities in critical thinking and learning.

Unearthing, understanding, and unpacking histories of colonial violence, and its manifestations that impact Latinx communities, necessitates that as educators we commit ourselves to the decolonization of knowledge, pedagogy, and practice. Relatedly, we must challenge hegemonic notions of rights and citizenship that compromise the humanity, dignity, and enfranchisement of communities. Indeed, we must develop decolonial pedagogies that are *responsible* and *responsive* to the traumas that our students carry. We must decolonize the university that often forecloses students of color, especially Latinx, from opportunities to meaningfully learn toward the development of a critical consciousness. As educators we can and must foster students’ radical hope, intersectional solidarity, and collective voice and action in times of difficulty when, as COVID-19 has well demonstrated, human rights continue to be woefully unfulfilled.

## Footnotes

1 We use “Latinx” to denote the complexities of *Latinidad*, that according to Alan Pelaez Lopez (2018) extend beyond gender inclusivity and language to uncover four interconnected wounds that shape Latinx subjectivities. These wounds, signaled by the “x,” include: the wound of settler colonialism, anti-Blackness, femicides, and inarticulation, or the inability to articulate and denounce intersecting forms of violence. Accordingly, we use the “x” to trouble the gendered construction of Latino or Latina/o a pan-ethnic/racial and transnational community with heritage in Latin America.
